# Specific Detection and Differentiation Between *Brucella melitensis* and *Brucella abortus* by a Duplex Recombinase Polymerase Amplification Assay

**DOI:** 10.3389/fvets.2020.539679

**Published:** 2020-11-25

**Authors:** M. M. Gumaa, Zhaocai Li, Xiaoan Cao, Nianzhang Zhang, Zhongzi Lou, Jizhang Zhou, Baoquan Fu

**Affiliations:** ^1^State Key Laboratory of Veterinary Etiological Biology, Key Laboratory of Grazing Animal Diseases of Ministry of Agriculture, Key Laboratory of Veterinary Public Health of Ministry of Agriculture, Lanzhou Veterinary Research Institute, Chinese Academy of Agricultural Sciences, Lanzhou, China; ^2^Kassala Veterinary Research Laboratory, Central Veterinary Research Laboratory, Animal Resources Research Corporation, Khartoum, Sudan

**Keywords:** Duplex RPA, *B. melitensis*, *B. abortus*, real-time PCR, AMOS multiplex PCR

## Abstract

Brucellosis is a highly contagious zoonosis caused by a species under the genus *Brucella*. A duplex recombinase polymerase amplification (Duplex RPA) assay for the specific detection of *Brucella melitensis* and *Brucella abortus* was developed in this study. Primers were designed targeting hypothetical protein genes and membrane transporter genes of *B. melitensis* and *B. abortus*, respectively. The newly developed assay was validated for its analytical sensitivity and specificity. Different samples were collected from the Qinghai, Inner Mongolia, and Xinjiang provinces. After DNA extraction, the samples were analyzed by Duplex RPA, real-time PCR, and multiplex AMOS PCR to estimate the prevalence of brucellosis in sheep and yak in West China. The analytical sensitivities of Duplex RPA were 9 × 10^2^ plasmid copies of *B. melitensis* and 9 × 10^1^ plasmid copies of *B. abortus*, but by mixing the reaction tubes after 4 min of incubation, the sensitivities were 4 × 10^0^ and 5 × 10^0^ copies of *B. melitensis* and *B. abortus*, respectively. There was no cross-reactivity with *Brucella suis, Chlamydia abortus, Salmonella typhimurium, Escherichia coli*, and *Toxoplasma gondii*. The screening of field samples by Duplex RPA revealed that the prevalence of *B. melitensis* in sheep and yak was 75.8% and the prevalence of *B. abortus* was 4.8%. Multiplex AMOS PCR showed that the prevalence of *B. melitensis* was 19.3%, and that of *B. abortus* was 4.8%. It was concluded that the developed Duplex RPA is sensitive and specific to the detection of and differentiation between *B. melitensis* and *B. abortus* which will be useful in epidemiological surveillance and in the clinical settings.

## Introduction

Brucellosis is a contagious zoonotic disease caused by different species under the genus *Brucella* (1). *Brucella melitensis, Brucella abortus*, and *Brucella suis* are responsible for severe human infection in addition to economic losses in livestock due to abortions and loss of fertility (2). Two species *B. melitensis* and *B. abortus* are considered the most important species that cause illness in humans and domestic animals in many countries throughout the world. Three biovars of *B. melitensis* mainly infect sheep and goats, while the preferred hosts of seven biovars of *B. abortus* are cattle and buffaloes (3). The main clinical manifestations of *B. melitensis* infection in sheep and goats and *B. abortus* in cattle are abortion and stillbirth in females and orchitis and loss of fertility in males (4). The distribution of *B. melitensis* has long been associated with the Mediterranean littoral, however, it is now known to be more widely distributed, with only North America, North Europe, South-East Asia and Oceania being spared (5). *B. abortus* is distributed in many African, European, Asian, and American countries (6). Identification of *Brucella* organisms at the species and biotype level, mainly relies on culture, isolation, and subsequent identification by morphology, biochemical tests, reaction with monospecific antisera, sensitivity to dyes, and phage lysis (7). Several molecular methods have been developed to detect *Brucella* at the species level despite the high sequence homology between different species (8). The first species-specific PCR to be developed was multiplex AMOS PCR which can detect *B. melitensis, B. abortus* biovar 1, 2, and 4, *B. ovis*, and *B. suis* biovar 1 (9). Later Bruce-Ladder multiplex PCR was developed to identify 10 species in addition to vaccine strains of *B. abortus* and *B. melitensis* (10). Real-time PCR and multiplex PCR microarrays have been established to identify species (11, 12). Real-time and lateral flow dipstick RPAs have been developed for the detection of *Brucella* spp. (13). Because of the high prevalence of *B. melitensis* and *B. abortus* in many countries throughout the world, their economic impact is due to losses of productive livestock. In addition to their zoonotic potential, we developed a novel Duplex RPA for the species specific detection of *B. melitensis* and *B. abortus* and to differentiate between them, and then validated the assay in field samples compared to real-time PCR and multiplex AMOS PCR. Another target of this work was to estimate the prevalence of brucellosis outbreaks in the Qinghai, Inner Mongolia, and Xinjiang provinces in West China by cross-sectional molecular detection by Duplex RPA, real-time PCR, and multiplex AMOS PCR.

## Materials and Methods

### Bacterial Strains

*B. melitensis biovar 3, B. melitensis biovar 1* M5 vaccine strain, *B. abortus* S19, and *B. suis biovar 1* S2 vaccine were used as reference strains. The Reference DNA was obtained from Lanzhou Veterinary Research Institute and Harbin Veterinary Research Institute and was confirmed by AMOS PCR (9). The concentration of *B. melitensis* biovar 3 DNA was 10 ng/μL, that of *B. melitensis* biovar 1 M5 strain was 13 ng/μL, that of *B. abortus* S19 was 14 ng/μL, and that of *B. suis* biovar 1 S2 vaccine strain was 11 ng/μL. The purity of DNA samples measured by the ratio A260/A280 was 1.8–2.0.

### Collection of Samples and DNA Extraction

Sixty-two different samples from sheep and yak were collected from the Qinghai, Inner Mongolia, and Xinjiang provinces. Tissue samples were collected in sterile plastic bags and milk samples were collected in sterile vials. The number and types of samples from each province are described in Table 1. Genomic DNA was extracted using the TIANamp Genomic DNA kit (TIANGEN Biotech, Beijing, China). Tissue samples were cut into small pieces and ground in a mortar and pestle. Then small portions of grinded tissues (>10 mg) were lysed by the addition of 200 μL of GA buffer, 20 μL of proteinase K (provided in the kit), and RNase (100 mg/mL), and were incubated in a water bath at 56°C for 20–30 min until completely lysed. The next steps were carried out following the kit manufacturer's instructions. Milk samples (10 mL) were centrifuged at 3,000 RPM for 5 min, and fluid between the supernatant and deposit was pipetted and discarded. The supernatant and deposit were mixed together, transferred to 1.5 mL tubes, and then lysed with the addition of 200 μL GA buffer, 20 μL proteinase K, and RNase (100 mg/mL), and were incubated at 56°C for 10 min. The remaining steps were performed as outlined in the kit manual. The extracted DNA samples were quantified using NanoDrop (Infinite 200 PRO, TECAN, Groedig, Austria). DNA samples were stored at −20°C until analysis.

**Table 1:** The results of Duplex RPA and multiplex AMOS PCR in field samples.

**Province**	**Sample types and number**	**Animal species**	**RPA**	**AMOS-PCR**
Qinghai	Liver (14)	Yak (aborted fetus)	*B. melitensis (5)*	–
	Intestine (3)	Yak (aborted fetus)	*B. melitensis (3)*	–
	Stomach (14)	Yak (aborted fetus)	*B. melitensis (15)*	–
	Lung (16)	Yak (aborted fetus)	*B. melitensis (14)*	***B. melitensis (1)***
	Intestine (3)	Sheep (aborted fetus)	*B. melitensis (1)*	–
			*B. abortus (2)*	***B. abortus (2)***
	Lung (2)	Sheep (aborted fetus)	*B. melitensis (1)*	–
	Liver (2)	Sheep (aborted fetus)	*B. abortus (1)*	***B. abortus (1)***
	Spleen (1)	Sheep (aborted fetus)	*B. melitensis* (1)	–
Inner Mongolia	Milk (17)	Sheep	*B. melitensis (18)*	***B. melitensis(****19****)***
Xinjian	Liver (20)	Sheep (aborted fetus)	*B. melitensis (20)*	–
	Lung (2)	Sheep (aborted fetus)	*B. melitensis (2)*	–
	Stomach (2)	Sheep (aborted fetus)	*B. melitensis (2)*	–
	Kidney (2)	Sheep (aborted fetus)	*B. melitensis (2)*	–
	Spleen (2)	Sheep (aborted fetus)	*B. melitensis (2)*	–
Total	62		*B. melitensis (47) (75.8%)*	*B. melitensis (21) (19.3%)*
			*B. abortus (3) (4.8%)*	*B. abortus (3) (4.8%)*

### Selection of Specific Sequences and Bioinformatics Analysis

According to the previous studies of (9, 15, 20), the specific sequence regions of *B. melitensis* (Accession number: CP007763.1 of *B. melitensis* 16 M strain) of the hypothetical protein gene and *B. abortus* (Accession number: AE017224.1 of *B. abortus* strain 9-941) of the membrane transporter gene was selected and retrieved from the NCBI GenBank database (http://www.ncbi.nih.gov). The similarities between each sequence and other sequences were determined by the Basic Local Aligned Sequence Tool (BLAST). Multiple sequence alignment of each *B. melitensis* and *B. abortus* sequence was carried out by the MegAlign software (DNASTAR Lasergene, Madison, Wisconsin USA).

### Primers Design

Primers were designed according to the appendix in the TwistAmp™ reaction kit manuals (http://www.twistdx.co.uk/images/uploads/docs/Appendix.pdf). Unlike PCR, the RPA primers should be 30–35 nucleotides in length, with GC content between 40 and 60%, no shorter than 30 nucleotides and longer than 45 nucleotides, no long tracks of guanines at the 5' end, Gibbs free energy (-_ΔG) should be between −4 and −5 kcal/mol for both the 5' and 3' ends of the primers. The species-specific primer pairs were designed by the Oligo Primers analysis software (Version 6.31 Molecular Biology Insights, Inc., USA), the parameters in the software such as primer length, and amplified sequence length range were adjusted, the primers were synthesized by (Tsingke Biological Technology, Xian, China).

### Duplex Recombinase Polymerase Amplification (Duplex RPA) Development and Primary Experimentation

**Figure 1:**
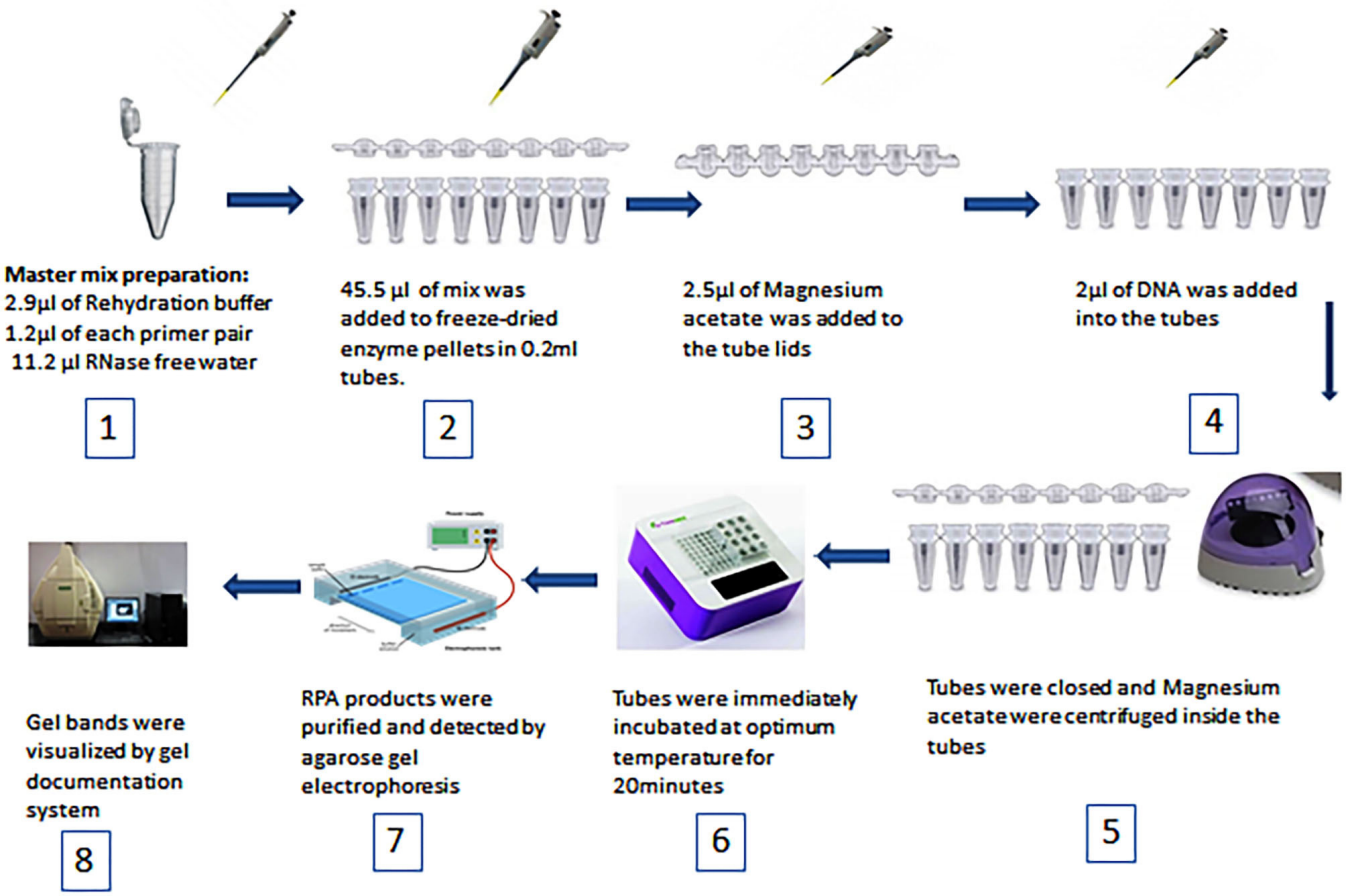
Schematic diagram of RPA steps and equipment used.

Duplex RPA was performed with a volume of 50 μL using a TwistAmp Basic kit (TwistDx, Cambridge, United Kingdom). The master mix was composed of 29.5 μL of RPA rehydration buffer, 1.2 μL (5 μM) of each primer, 11.2 μL of RNase free water (TAKARA Clontech, Shiga, Japan), 2.5 μL (280 mM) of magnesium acetate, and 2 μL of the DNA template. All reagents were prepared in 1.5 mL tubes except magnesium acetate and the DNA template. Then 45.5 μL of master mix was added to freeze-dried enzyme pellets in 0.2 mL reaction tubes each containing dried enzyme pellets. Magnesium acetate was pipetted into the tube lids. Subsequently 2 μL of the standard or genomic DNA template was added to each tube. The tubes were closed, the magnesium acetate was centrifuged into the tubes using a Minispin centrifuge, and the tubes were immediately placed in a dry bath heat block (TIANGEN Biotech, Beijing, China). To select the optimum temperature, the reaction tubes were incubated at 37, 38, and 39°C for 20 min. After incubation, the RPA reaction products were purified by the TIANquick Midi Purification Kit (TIANGEN Biotech, Beijing, China). The purification procedure was carried out according to the steps outlined in the kit manual. Purified RPA products were loaded in a 1.5–2% agarose gel and agarose gel electrophoresis was carried out for 15–30 min. The specific bands were visualized by a gel documentation system (BIORAD, Hercules, California, USA). The steps of RPA are demonstrated in Figure 1. The total time of the RPA reaction was between 45 and 60 min.

To examine candidate primers and optimal temperature, genomic DNA of *B. melitensis* biovar *3* and *B. abortus strain 19* were used as positive control and *B. suis* biovar 1 S2 vaccine was used as negative control in addition to RNase free water as a non-template control.

### Construction of Positive Control Plasmid DNA

The positive control plasmid DNA was constructed by ligation of a specific sequence of *B. melitensis* and *B. abortus* to the plasmid vector pMD-19 (TAKARA Clontech, Shiga, Japan). After purification of the RPA product, agarose gel electrophoresis, and the visualization of the bands, specific bands of *B. melitensis* and *B. abortus* were cut and subjected to gel DNA extraction by an AxyPrep DNA gel extraction kit (Axygen Bioscience, California, USA). After extraction from the gel, DNA was ligated to the plasmid vector by the addition of 4 μL of DNA to 1 μL of vector and 5 μL of 2 × solution (provided with plasmid vector) and then incubated at 16°C overnight. The transformation was performed after the incubation of DNA, plasmid vector, and 2 × solution mixtures by the addition of 5 μL of mixture to 50 μL of *Escherichia coli* DH5α competent cells in a 1.5 mL tube. The tube was then incubated on ice for 30 min, placed in a water bath at 42°C for 60–90 s, and placed again on ice for 3 min. One milliliter of Luria-Bertani (LB) broth was added to the tube which was then incubated in a shaker incubator at 37°C for 45 min. Luria Bertani (LB) agar plates containing 100 μg/mL ampicillin were inoculated with 100 μL of the cultured LB broth. The plates were incubated at 37°C for 18–24 h. Individual colonies (10, 13) were cultured into 1 mL of LB broth and incubated at 37°C in a shaker incubator. The plasmid DNA was extracted from the cultured broth by the TIAN prep Rapid Mini Plasmid Kit (TIANGEN Biotech, Beijing, China). The extracted plasmid was quantified by NanoDrop (Infinite 200 PRO, TECAN, Groedig, Austria). The copy numbers were calculated by the equation: number of copies = (amount ^*^ 6.022 × 10^23^)/(length ^*^ 1 × 10^9^^*^ 650). The quantified plasmid was serially diluted 10-fold with elution buffer supplied in the extraction kit and analyzed by RPA to determine analytical sensitivity (detection limit).

### Analytical Sensitivity of Duplex RPA

To determine the analytical sensitivity (detection limit) of Duplex RPA, serial dilutions of the constructed plasmid of *B. melitensis* ranging from 9 × 10^7^ and 9 × 10^1^ copy numbers per reaction and *B. abortus* ranging from 9 × 10^6^ and 9 × 10^1^ copy numbers per reaction were analyzed at 38°C for 20 min. Two microliters of each dilution were added to a single 0.2 mL tube, and in the last one 2 μL of RNase free water was added instead of the DNA template, which was considered a non-template control. Every run was repeated three times. To increase the sensitivity of Duplex RPA, further evaluation was performed by testing other 10-fold dilutions of the constructed plasmids of *B. melitensis* ranging from 4 × 10^5^ and 4 × 10^0^ copy numbers per reaction and *B. abortus* ranging from 5 × 10^6^ and 5 × 10^0^ per reaction. Each 0.2 mL tube received 2 μL of DNA of each dilution and the last tube received 2 μL of RNase free water added to be considered as a non-template control. The reaction tubes (0.2 mL) were removed from the dry bath heat block after 4 min from the beginning and were mixed by inversion 10 times and then placed back into the dry bath heat block. The reaction tubes were incubated at 38°C for 20 min. Every run was repeated three times.

### Analytical Specificity of Duplex RPA

To determine the analytical specificity, 2 μL of DNA of *B. melitensis* and *B. melitensis M5, B. abortus* S19, *B. abortus*, and *B. suis S2* was first confirmed by AMOS PCR (9) and analyzed by Duplex RPA at an optimal temperature for 20 min. Other DNA samples (2 μL) of organisms such as *Chlamydia abortus, Salmonella typhimurium, E. coli*, and *Toxoplasma gondii* were analyzed by Duplex RPA to determine analytical specificity.

### Real-Time PCR

The primer pair and probe of the real-time PCR assay were published by (14). Real-time PCR was prepared in a total volume of 20 μL. The total mix contained 10 μL of TaqMan Master mix (AceQs qPCR Probe Master Mix, Vanzyme, Nanjing, China), 0.4 μL of each primer, 0.2 μL of probe, 7 μL of RNase free water, and 2 μL of the DNA template. The reaction conditions were 95°C for 5 min followed by 40 cycles of 95°C for 10 s and 60°C for 30 s. The reaction was incubated in a real-time PCR thermocycler (BIORAD, Hercules, California, USA). The primer pair and probe used in the real-time PCR are presented in Table 2.

**Table 2:** *B. melitensis, B. abortus*, and AMOS PCR primers.

**Primers pairs**	**Primers sequence**	**Product size**	**Reference**
*B. melitensis*	FP-5′ AAA ACA TTG ACC GCA TTC ATG GGC TTC GTC 3′ RP-5′ CAA TTA TCG CTG TCA CTG TTGCAA GTA TGG 3′	167 bp	This study
*B. abortus*	FP-5′ GACAAGGTGTATATCAACCAGCAGGTCAAC 3′ RP-5′ GACCCTTCCCACCGCCAAAGACCGCAAACG 3′	235 bp	This study
Real-time PCR	IS421: cgctcgcgcggtggat IS511:cttgaagcttgcggacagtcacc ISTq:FAMacgaccaagctgcatgctgttgtcgatg-TAMRA	178 bp	Bounaadja et al. (14)
AMOS-PCR primers	Primer sequence(5′-3′)		
	AMOS-(A1) gacgaacggaatttttccaatccc	498 bp	Bricker and Halling (9)
	AMOS-(M) aaatcgcgtccttgctggtctga	730 bp	
	AMOS-(O) cgggttctggcaccatcgtcg	976 bp	
	AMOS-(S) gcgcggttttctgaaggttcagg	285 bp	
	AMOS-(IS711) tgccgatcacttaagggccttcat		

### Multiplex AMOS PCR

The AMOS (abortus-melitensis-ovis-suis) (9, 16) PCR was performed using five sets of primers (Table 2). The total reaction mix consisted of: 25 μL of 2XTaq master mix (TIANGEN Biotech, Beijing, China), 0.2 μM of each primer and RNase free water (TAKARA Clontech, Shiga, Japan), and 2 μL of the DNA template. The PCR conditions were as follows: initial denaturation at 96°C for 5 min followed by 40 cycles of 95°C denaturation for 1 min, 55.5°C annealing for 2 min and 72°C extension for 2 min, and a final extension at 72°C for 5 min. Species-specific bands were visualized after agarose gel electrophoresis.

### Screening of Field Samples With Duplex RPA and AMOS PCR

The collected field samples were simultaneously screened by Duplex RPA, real-time PCR, and AMOS PCR for the species-specific detection of *B. melitensis* and *B. abortus*. RPA and PCR procedures were performed as described above.

### Statistical Analysis

The results of the field sample screening by Duplex RPA and real-Time PCR were analyzed by Microsoft Excel version 2010 to estimate the prevalence of brucellosis in aborted and seropositive animals. The upper and lower limits of prevalence at 95% confidence intervals were calculated by Wilson score intervals using the online calculator available on the website: http://epitools.ausvet.com.au/content.php?page=CIProportion.

## Results

### Detection of Bacterial Strains by AMOS PCR

**Figure 2:**
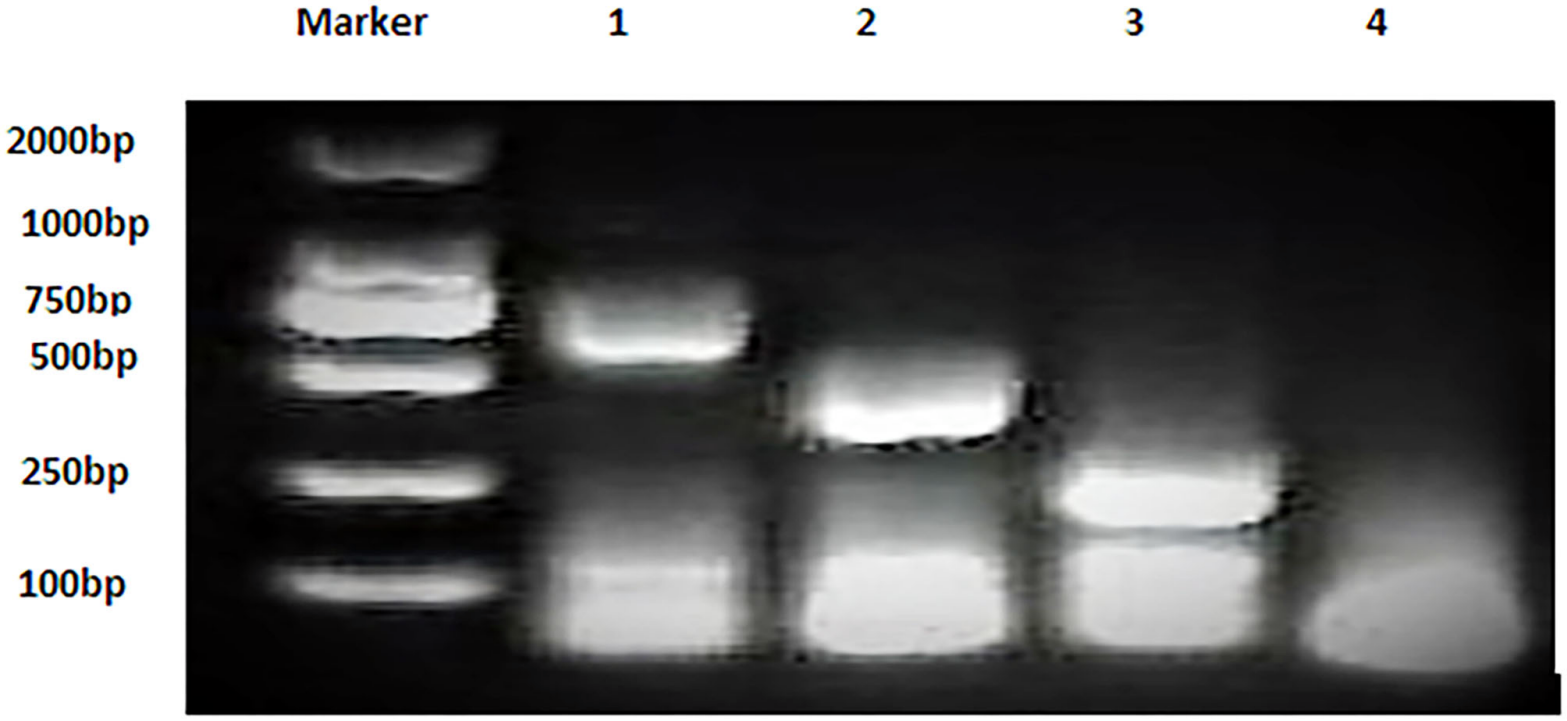
Multiplex AMOS PCR of *Brucella* spp. strains. Lane 1: *B. melitensis* 730 bp, Lane 2: *B. abortus* 498 bp, Lane 3: *B. suis* 285 bp, and Lane 4: non-template control RNase free water.

Bacterial strains showed positive AMOS PCR results characterized by a specific band for each species (Figure 2). *B. melitensis* yielded a band of 730 bp, *B. abortus* had a 498 bp band, and *B. suis* produced a 285 bp band.

### Sequence Alignments and Primer Pairs

**Figure 3:**
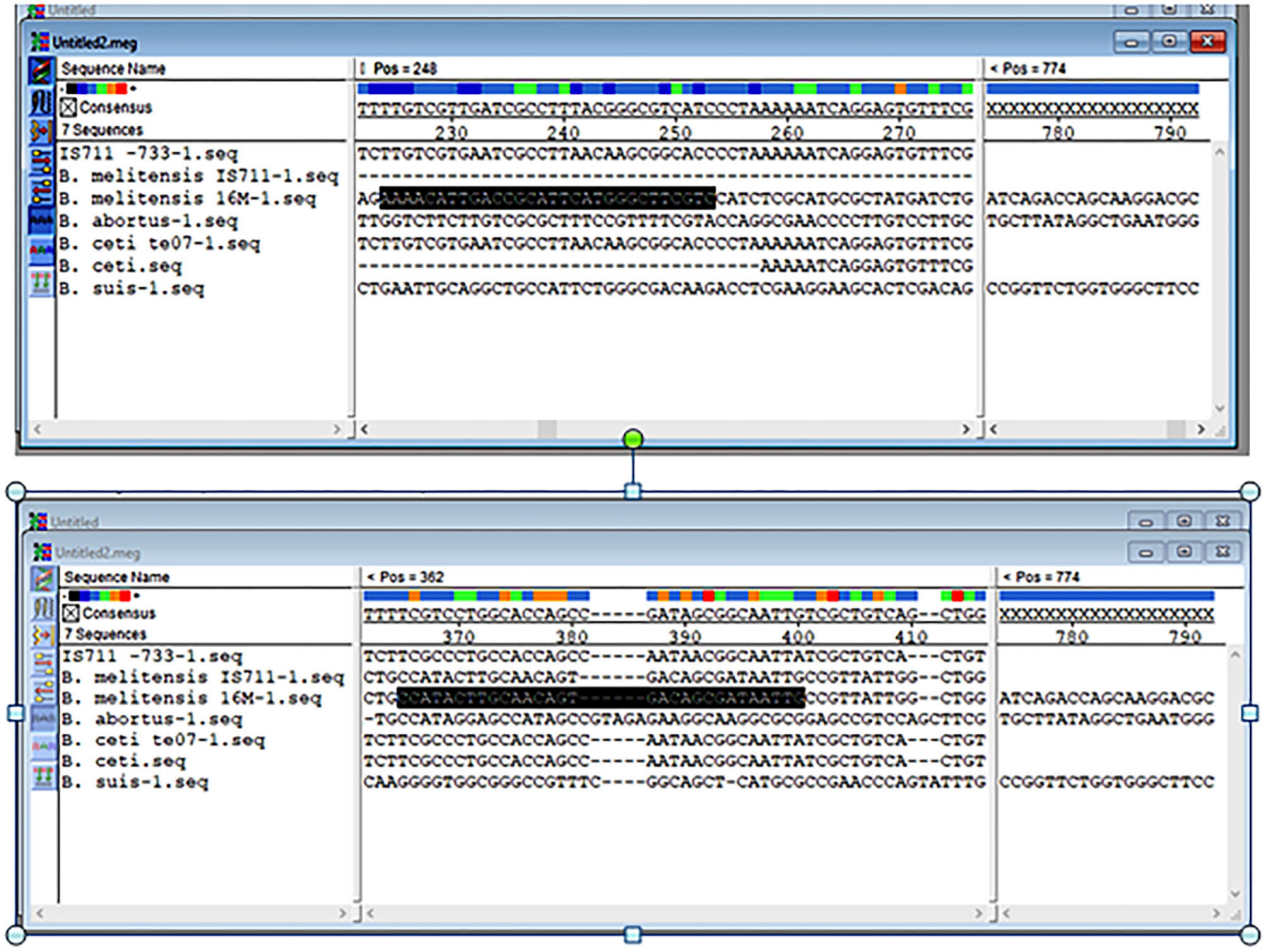
Alignments of the partial sequence of *B. melitensis* IS711 and hypothetical protein coding region. The multiple sequences have been aligned by the Clustal W method.

**Figure 4:**
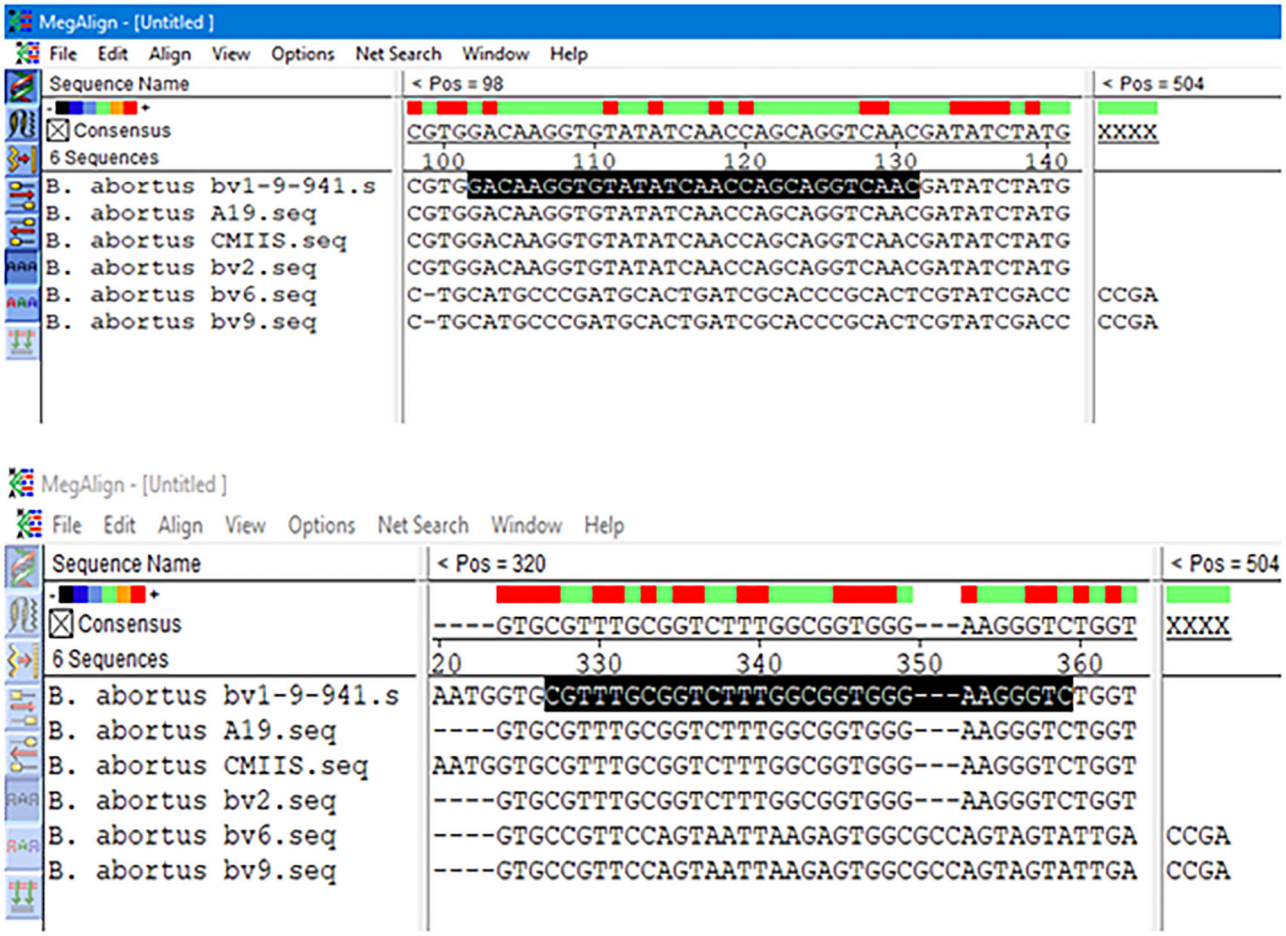
Alignments of *B. abortus* outer membrane transporter gene coding region different partial sequences. The alignment tool was Clustal W.

The results obtained from BLAST revealed the high similarity of each sequence of *B. melitensis* and *B. abortus* to different strains of *B. melitensis* and *B. abortus*. The selected *B. melitensis* sequence showed some similarity with *Brucella ceti* and *B. suis*. Thus, the selected primers were located in the *B. melitensis* specific region. The selected sequence region of *B. abortus* did not show any similarities with other species. The alignment of partial sequences from *B. melitensis* is demonstrated in Figure 3. The partial sequence of *B. abortus* and its similar sequence alignments are shown in Figure 4. The primer pairs of *B. melitensis* and *B. abortus* are presented in Table 1. Forward and reverse primer locations are indicated in each sequence of *B. melitensis* and *B. abortus* by black highlights in Figures 3, 4.

### Duplex RPA Optimal Conditions

Duplex RPA can be performed at reaction temperatures ranging from 37 to 39°C. There was no difference in the results between 37, 38, and 39°C. The selected reaction conditions were 38°C for 20 min. The selected temperature was arbitrarily selected. The selected primers of *B. melitensis* yielded a 167 bp band, while *B. abortus* yielded a 235 bp band.

### Analytical Sensitivity and Specificity

**Figure 5:**
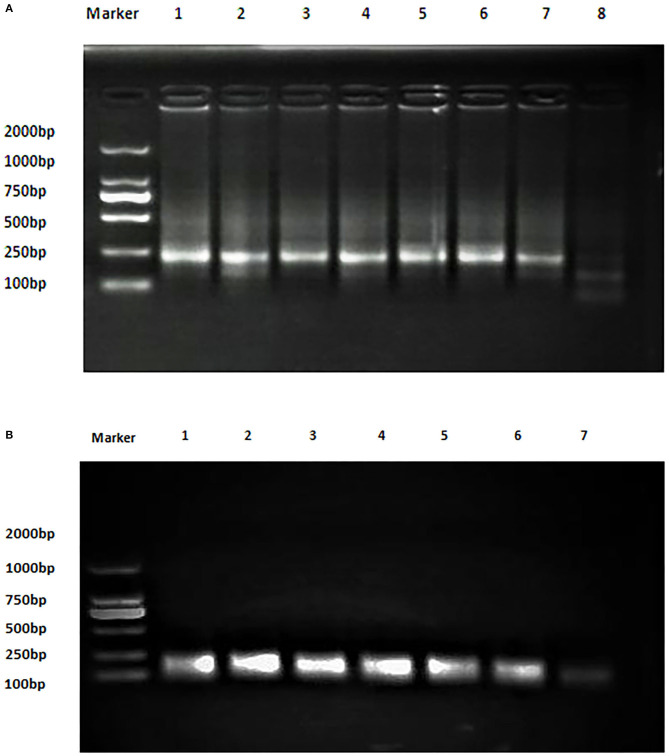
Analytical sensitivity of *B. melitensis* and *B. abortus* RPA: **(A)** *B. abortus melitensis* plasmid serial dilutions, DNA marker 2,000 bp. Lane 1: 9 × 10^7^, Lane 7: 9 × 10^1^ considered negative. **(B)** *B. abortus* plasmid serial dilutions, DNA marker 2,000 bp, Lane 1: 9 × 10^6^ and Lane 7 is the non-template control (RNase free water).

**Figure 6:**
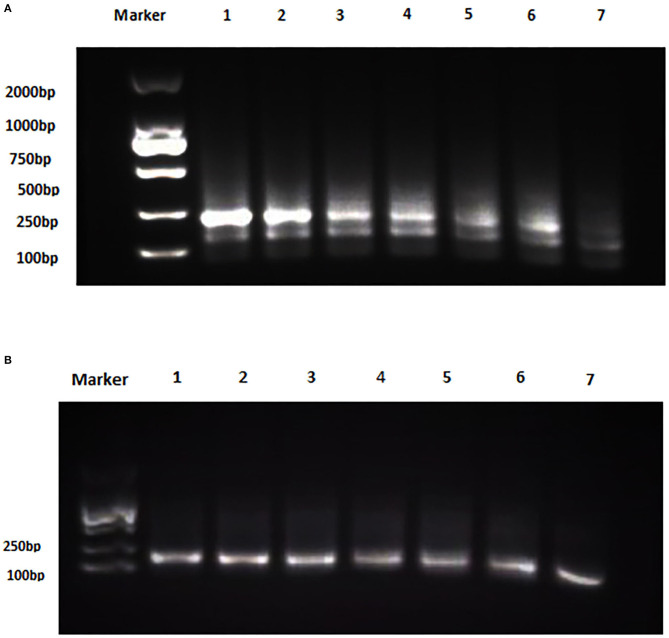
Analytical sensitivity of *B. melitensis* and *B. abortus* RPA: **(A)** *B. melitensis* plasmid serial dilutions, DNA marker 2,000 bp. Lane 1: 4 × 10^5^, Lane 6: 4 × 10^0^, and Lane 7 is the non-template control (RNase free water). **(B)** *B. abortus* plasmid serial dilutions, DNA marker 2,000 bp, Lane 1: 5 × 10^5^ and Lane 7: 5 × 10^0^ , and Lane 8 is the non-template control (RNase free water).

**Figure 7:**
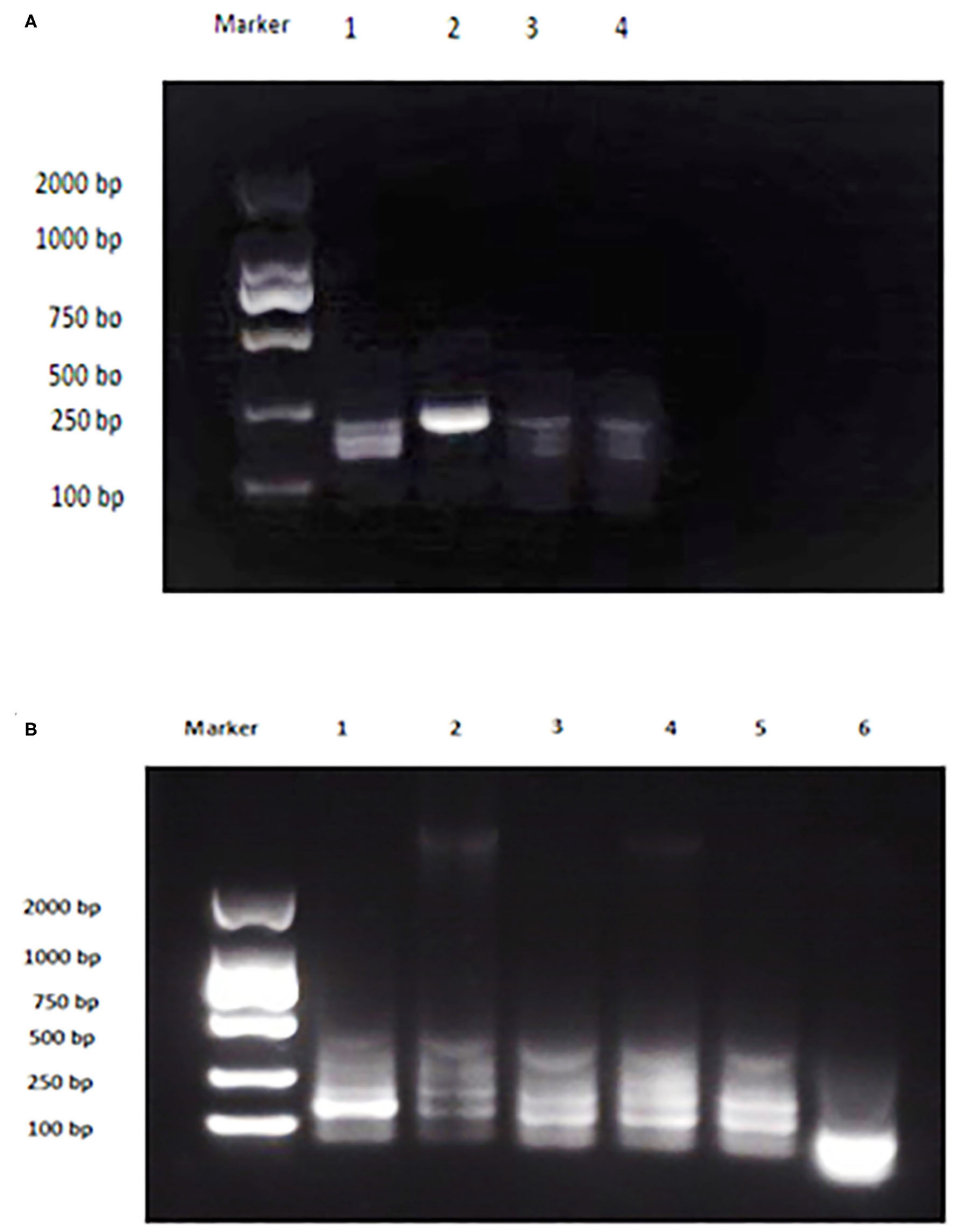
Analytical specificity of Duplex RPA. **(A)** DNA marker 2,000 bp, Lane 1: *B. melitensis bv. 3*, Lane 2: *B. abortus* A 19, Lane 3: *B. suis* S2, Lane 4: non-template control. **(B)** DNA marker 2,000 bp, Lane 1: *B. melitensis* M5, Lane 2: *C. abortus*, Lane 3: *S. typhimurium*, Lane 4: *E. coli*, Lane 5: *T. gondii*, Lane 6: non-template control (RNase free water).

The analytical sensitivity of Duplex RPA by amplifying different serial dilutions of plasmids bearing target sequences of *B. melitensis* and *B. abortus* was 9 × 10^2^ copies of the constructed plasmid of *B. melitensis* and 9 × 10^1^ copies of *B. abortus* plasmid (Figures 5A, 6A). The test could detect *B. melitensis biovar 3, B. melitensis* M5, and *B. abortus* A19 (Figures 6, 7A). The mixing of the RPA reaction 4 min after starting increased the sensitivity to 4 × 10^0^ and 5 × 10^0^ copies of *B. melitensis* and *B. abortus*, respectively (Figures 5B, 6B). There was no cross-reactivity of the developed RPA with *B. suis* S2 and other bacteria, including *C. abortus, S. typhimurium, E. coli*, and the parasite *T. gondii* (Figure 7B).

### Results of Screening Field Samples

Different types of sheep and yak samples from the Qinghai, Inner Mongolia, and Xingjian provinces showed a high prevalence of brucellosis, with prevalence rates of 80.6% by duplex RPA and 95.2% by real-time PCR (Table 3). The incidence of *B. melitensis* was 47 (75.8%), and that of *B. abortus* was 3 (4.8%). The incidence results obtained by AMOS PCR were 12 (19.3%) for *B. melitensis* and 3 (4.8%) for *B. abortus* (Table 1). A sheep was found to be infected with *B. melitensis* and *B. abortus*, and yak were found to be infected with *B. abortus* and *B. melitensis* (Table 1). These results reveal the transmission of *B. abortus* to sheep and *B. melitensis* to yak.

**Table 3:** The results of Duplex RPA and real-time PCR in field samples.

**Province**	**Total number of samples**	**Animal species**	**Duplex RPA**	**CI 95%**	**Real-time PCR**	**CI 95%**
			**+**		**+**	
Qinghai	22 tissue samples	Yak	19 (86.4%)	0.7–0.95	21 (95.4%)	0.78–0.99
	8 tissue samples	Sheep	6 (75%)	0.41–0.93	8 (100%)	0.67–1.00
Inner Mongolia	20 milk	Sheep	13 (65%)	0.43–0.82	20 (100%)	0.84–1.00
Xinjiang	12 tissue samples	Sheep	12 (100%)	0.76–1.00	10 (83.3%)	0.55–0.95
Total	62		50 (80.6%)	0.7–0.9	59 (95.2%)	0.87–0.98

## Discussion

Brucellosis is a re-emerging zoonotic disease caused by the closely related species of the genus *Brucella*. *B. melitensis* and *B. abortus* were the first described species in 1887 and 1895 A.D., respectively (22). Livestock species (cattle, sheep, goats, swine, and camel) can be infected with *B. melitensis, B. abortus*, and *B. suis*, which are responsible for severe human infection (23). Cattle can be infected by *B. abortus* and transiently infected with *B. suis* and more commonly by *B. melitensis* when they come into direct contact with infected pigs, goats, and sheep in common pastures and at shared water sources. *B. abortus, B. melitensis*, and *B. suis* can be transmitted by cow's milk and cause a serious public health threat (24). The newly developed Duplex RPA could detect either *B. melitensis* or *B. abortus* with a reaction time of 20 min; additionally it did not require sophisticated equipment instead employing a water bath or a heat block dry bath. The previously developed types of PCR, i.e., AMOS PCR, Bruce-Ladder PCR, and multiplex real-time PCR, were successfully capable of detecting and differentiating between different species under the genus *Brucella*. Furthermore, these techniques can differentiate between field strains and vaccine strains (10, 19), but these techniques require 2–4 h of reaction time and require a thermocycler. Similar to PCR and multiplex PCR, Duplex RPA was used for end-point detection by agarose gel electrophoresis, which increased the time of the test. Therefore, the developed Duplex RPA cannot be considered a rapid test. Other types of PCR have been developed for species-specific detection and differentiation between field and vaccine strains (*B. melitensis, B. suis*, and *B. abortus*-specific PCR) (18, 21, 25). One of the limitations of developed RPA is the inability to differentiate vaccinated animals from naturally infected animals. Generally, RPA is considered a newly invented technique that can rapidly amplify DNA or RNA within 10–20 min (26). Similar to PCR and real-time PCR, multiplexing in RPA is applicable. Therefore, many sensitive and specific multiplexed RPA assays have been developed for the diagnosis of many pathogens (27). The sensitivity of RPA can be increased by mixing during incubation, which is supported by a selection of short fragments of the target sequence that leads to swift amplification (28, 29). In multiplex lateral flow RPA for the detection of many intestinal protozoa, the molecular sensitivities are 403 synthetic gene copies per reaction of the Giardia, 425 of the Cryptosporidium, and 368 of the Entamoeba target (30). The developed multiplex RPA for the detection of methicillin-resistant *Staphylococcus aureus, Salmonella enterica*, and *Neisseria gonorrhoeae* showed limits of detection of 10 colony-forming units for methicillin-resistant *S. aureus* and *S. enterica* and 100 colony-forming units for *N. gonorrhoeae* (30). The detection sensitivity of multiplex RPA for the detection of *Campylobacter coli and Campylobacter jejuni* was 1 CFU/reaction in pure culture. In the case of food applications, the detection limit of *C. coli and C. jejuni* using the RPA assay were 1 CFU/mL from chicken broth, 10^3^ CFU/g from egg and chicken meat samples without a pre-enrichment procedure, and 1 CFU/g from 24 h enriched egg and chicken meat samples (17). Similar to the previously developed multiplex RPA assays, the developed Duplex RPA is highly sensitive especially after mixing components after 4 min of incubation. However, the end-point detection of most of the multiplex RPA assays depend on real-time fluorescence detection or lateral flow immunochromatographic strips, which make it a rapid technique.

In western China, bacterial isolation and identification, AMOS PCR and multi-locus variable number tandem repeat analysis found that *B. melitensis* biovar *3* was the dominant causative agent of sheep brucellosis, while *B. abortus* biovars 1 and 3 were found in infected yaks (31, 32). This study agrees with a previous study reporting the isolation of *B. melitensis* from yak in Qinghai province, west of China (33). The importance of *Brucella* species identification, in addition to the determination of the epidemiological situation, is also essential for vaccine-type selection, for example; if *B. melitensis* is endemic to a region, *B. melitensis* vaccines such as *B. melitensis* Rev.1 or *B. melitensis* M5 can be used; in contrast, *B. abortus* strain 19 can only be used areas in which *B. abortus* is endemic. There was high variation between the results obtained by RPA and AMOS PCR, which may be attributed to the higher sensitivity of RPA and its ability to amplify DNA in the presence of PCR inhibitors (26); conversely, AMOS PCR amplifies long fragments DNA of *B. melitensis* and *B. abortus* which are 730 bp and 498 bp, respectively, influencing the sensitivity (34). In addition, AMOS multiplex PCR can only detect all biovars of *B. melitensis*, biovars 1, 2, and 4 of *B. abortus, B. suis* biovar 1, and *B. ovis* (9). The cost estimation of one reaction of RPA is 8 Euros while, that of AMOS PCR is 1 Euro and that of real-time PCR is 3 Euros. The high cost of the RPA reaction is attributed to the production of RPA reagents by one company (35).

From previous studies, the species and biovars isolated from western China were *B. melitensis* biovars 1 and 3, *B. abortus* biovars 1 and 3, and *B. suis* biovar 3 (33), which explains the variation of the results between AMOS-PCR, real-time PCR, and Duplex RPA. Through our work, RPA products can be purified, sequenced, or applied for ligation to plasmid vectors and cloning. This study has limitations, mainly in analyzing an inadequate number of *Brucella* species and strains and other genetically related bacteria. Therefore, this work must be continued by analyzing other species and biovars of *B. melitensis, B. abortus*, and *B. suis*. Further, this study can be considered as preliminary and key for the development of real-time or lateral flow RPA for the detection of two or three species of *Brucella* or the differentiation between field and vaccine strains. Additionally, RPA can be developed for the simultaneous detection of many bacteria species that can cause abortion, such as *C. abortus, Coxiella burnetti*, and *Brucella* spp.

It is concluded that Duplex RPA is an isothermal assay for specific detection and differentiation between *B. melitensis* and *B. abortus* that is highly sensitive and specific. The assay takes less time (20 min incubation) than multiplex AMOS PCR and real-time PCR. The test sensitivity can be increased by mixing the reaction components. The developed RPA is more sensitive than multiplex AMOS-PCR. Because the test still has limitations characterized by using agarose gel electrophoresis, this could further be improved by the application of a real-time exoprobe and a lateral flow dipstick for end-point detection. These characteristics demonstrate that Duplex RPA is a sensitive, specific, and direct detection technique for the species-specific detection of brucellosis.

## Data Availability Statement

The original contributions presented in the study are included in the article/Supplementary Material, further inquiries can be directed to the corresponding author/s.

## Ethics Statement

All animals were handled, samples collected and processed, and all techniques carried out in strict accordance with good animal practice according to the Animal Ethics Procedures and Guidelines of the People's Republic of China. The Animal Administration and Ethics Committee of Lanzhou Veterinary Research Institute, Chinese Academy of Agricultural Sciences approved the study (Permit No. LVRIAEC-2014-009). Written informed consent was obtained from the owners for the participation of their animals in this study.

## Author Contributions

GM, XC, JZ, and BF designed the study and wrote the paper. GM performed all the experiments, ZLi performed PCR, real-time PCR, and results analysis. ZLo participated in the collection and preparation of samples. NZ participated in article editing and proofreading. All authors contributed to the article and approved the submitted version.

## Conflict of Interest

The authors declare that the research was conducted in the absence of any commercial or financial relationships that could be construed as a potential conflict of interest.
