# Supplementary material for: Specific Detection and Differentiation Between Brucella melitensis and Brucella abortus by a Duplex Recombinase Polymerase Amplification Assay
Source: Front Vet Sci. 2020 Nov 25;7:539679. doi: 10.3389/fvets.2020.539679 (PMC7732630; doi:10.3389/fvets.2020.539679)
Supplement: Supplementary file 1 [file Data_Sheet_1.docx]

Table S1: Detailed collected samples of three provinces and Real time PCR results by ct values, Duplex RPA and AMOS-PCR of tissue samples of aborted fetuses of sheep and Yak from Qinghai province:

| No. | Sample type | Spp. | Ct/min | Results | RPA | AMOS-PCR |
| --- | --- | --- | --- | --- | --- | --- |
| 1 | liver | Yak | 32.28 | + | B. melitensis | - |
| 2 | intestine | Yak | 18.16 | + | B. melitensis | - |
| 3 | Heart/lung | Yak | 22.39 | + | B. melitensis | - |
| 4 | Liver | Yak | 23.72 | + | B. melitensis | - |
| 5 | Heart/lung | Yak | 24.15 | + | B. melitensis | - |
| 6 | Stomach | Yak | 22.51 | + | B. melitensis | - |
| 7 | Heart/lung | Yak | 32.12 | + | B. melitensis | - |
| 8 | Stomach /intestine | Yak | 29.44 | + | B. melitensis | - |
| 9 | Liver | Yak | 31.88 | + | B. melitensis | - |
| 10 | Stomach | Yak | 29.30 | + | B. melitensis | - |
| 11 | Heart/ lung | Yak | 30.18 | + | B. melitensis | B. melitensis |
| 12 | Spleen | Yak | 30.66 | + | B. melitensis | - |
| 13 | Heart/lung | Yak | 21.30 | + | B. melitensis | - |
| 14 | Liver | Yak | 28.67 | + | B. melitensis | - |
| 15 | Intestine | Yak | 30.29 | + | B. melitensis | - |
| 16 | Liver | Yak | 30.14 | + | B. melitensis | - |
| 17 | Lung | Yak | 28.34 | + | B. melitensis | - |
| 18 | Stomach | Yak | No | - | B. melitensis | - |
| 19 | Spleen | Sheep | 32.57 | + | B. melitensis | - |
| 20 | Lung | Sheep | 29.01 | + | B. melitensis | - |
| 21 | Intestine | Sheep | 30.16 | + | B. melitensis | - |
| 22 | Intestine | Sheep | 30.15 | + | B. abortus | B. abortus |
| 23 | Liver | Sheep | 31.41 | + | - | - |
| 24 | Stomach | Yak | 25.99 | + | - | - |
| 25 | Heart/lung | Sheep | 31.26 | + | B. melitensis | - |
| 26 | Liver | Sheep | 32.43 | + | B. abortus | - |
| 27 | Intestine | Sheep | 31.17 | + | B. abortus | B. abortus |
| 28 | Liver | Yak | 21.03 | + | - | - |
| 29 | Lung | Yak | 22.60 | + | - | B. abortus |
| 30 | Stomach | Yak | 24.05 | + | - | - |

Table S2: Real time PCR results by ct values, Duplex RPA and AMOS-PCR of milk samples collected from sheep in Inner Mongolia:

| No. | Sample type | Spp. | Ct/min | Results | RPA | AMOS- PCR |
| --- | --- | --- | --- | --- | --- | --- |
| 1 | Milk | Sheep | 22.85 | + | - | - |
| 2 | Milk | Sheep | 21.96 | + | - | - |
| 3 | Milk | Sheep | 23.04 | + | - | - |
| 4 | Milk | Sheep | 21.06 | + | - | - |
| 5 | Milk | Sheep | 24.20 | + | - | - |
| 6 | Milk | Sheep | 27.61 | + | B. melitensis | - |
| 7 | Milk | Sheep | 28.65 | + | B. melitensis | B. melitensis |
| 8 | Milk | Sheep | 22.18 | + | - | B. melitensis |
| 9 | Milk | Sheep | 21.77 | + | B. melitensis | B. melitensis |
| 10 | Milk | Sheep | 23.82 | + | B. melitensis | B. melitensis |
| 11 | Milk | Sheep | 16.74 | + | - | B. melitensis |
| 12 | Milk | Sheep | 26.29 | + | B. melitensis | B. melitensis |
| 13 | Milk | Sheep | 22.44 | + | B. melitensis | - |
| 14 | Milk | Sheep | 32.26 | + | B. melitensis | B. melitensis |
| 15 | Milk | Sheep | 30.19 | + | B. melitensis | B. melitensis |
| 16 | Milk | Sheep | 33.31 | + | B. melitensis | - |
| 17 | Milk | Sheep | 25.05 | + | B. melitensis | - |
| 18 | Milk | Sheep | 24.25 | + | B. melitensis | B. melitensis |
| 19 | Milk | Sheep | 25.92 | + | B. melitensis | B. melitensis |
| 20 | Milk | Sheep | 25.40 | + | B. melitensis | B. melitensis |

Table S3: Real time PCR results ct values, Duplex RPA and AMOS-PCR of tissue samples collected from aborted fetuses in Xinjiang Province:

| No | Sample type | Spp. | Ct/min | Results | RPA | AMOS-PCR |
| --- | --- | --- | --- | --- | --- | --- |
| 1 | Lung | Sheep | 26.36 | + | B. melitensis | - |
| 2 | Liver | Sheep | 34.99 | + | B. melitensis | - |
| 3 | Stomach | Sheep | 27.97 | + | B. melitensis | - |
| 4 | Kidney | Sheep | 24.06 | + | B. melitensis | - |
| 5 | Spleen | Sheep | 32.80 | + | B. melitensis | - |
| 6 | Liver | Sheep | No | - | B. melitensis | - |
| 7 | Kidney | Sheep | No | - | B. melitensis | - |
| 8 | Liver | Sheep | 28.35 | + | B. melitensis | - |
| 9 | Lung | Sheep | 31.43 | + | B. melitensis | - |
| 10 | Spleen | Sheep | 20.44 | + | B. melitensis | - |
| 11 | Stomach | Sheep | 21.67 | + | B. melitensis | - |
| 12 | Liver | Sheep | 26.79 | + | B. melitensis | - |

Note: Ct: cycle threshold.
