# Supplementary material for: Specific Detection and Differentiation Between Brucella melitensis and Brucella abortus by a Duplex Recombinase Polymerase Amplification Assay
Source: Front Vet Sci. 2020 Nov 25;7:539679. doi: 10.3389/fvets.2020.539679 (PMC7732630; doi:10.3389/fvets.2020.539679)

**Appendix2: The map of China showing the provinces from which the samples were collected: Red dots showing the province location.**


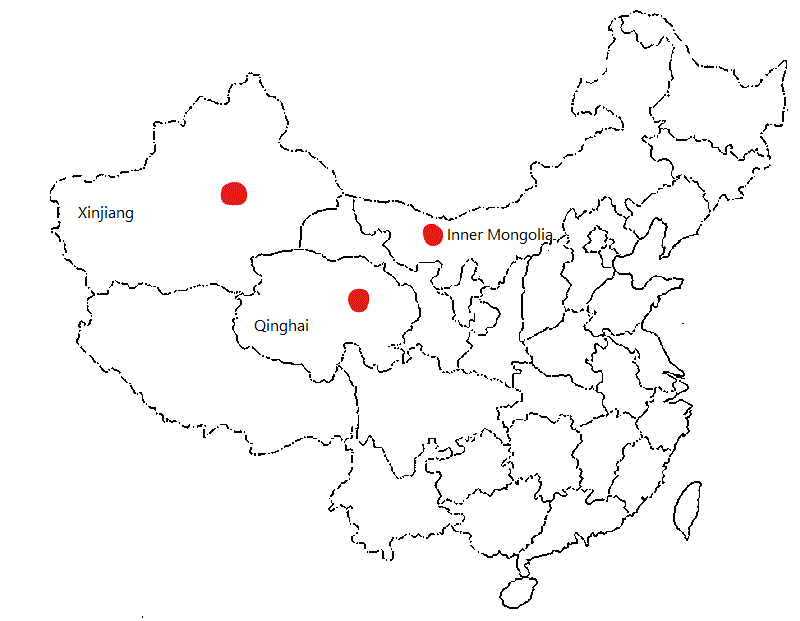

Supplement: Supplementary file 2 [file Data_Sheet_2.docx]
